# Identification of hospital cost drivers using sparse group lasso

**DOI:** 10.1371/journal.pone.0204300

**Published:** 2018-10-10

**Authors:** Piotr Swierkowski, Adrian Barnett

**Affiliations:** 1 AusHSI – Australian Centre for Health Services Innovation, Institute of Health Biomedical Innovation, Queensland University of Technology, Brisbane, Queensland, Australia; 2 Sunshine Coast Hospital and Health Service, Queensland Health, Queensland, Australia; Universitatsmedizin Greifswald, GERMANY

## Abstract

Public hospital spending consumes a large share of government expenditure in many countries. The large cost variability observed between hospitals and also between patients in the same hospital has fueled the belief that consumption of a significant portion of this funding may result in no clinical benefit to patients, thus representing waste. Accurate identification of the main hospital cost drivers and relating them quantitatively to the observed cost variability is a necessary step towards identifying and reducing waste. This study identifies prime cost drivers in a typical, mid-sized Australian hospital and classifies them as sources of cost variability that are either warranted or not warranted—and therefore contributing to waste. An essential step is dimension reduction using Principal Component Analysis to pre-process the data by separating out the low value ‘noise’ from otherwise valuable information. Crucially, the study then adjusts for possible co-linearity of different cost drivers by the use of the sparse group lasso technique. This ensures reliability of the findings and represents a novel and powerful approach to analysing hospital costs. Our statistical model included 32 potential cost predictors with a sample size of over 50,000 hospital admissions. The proportion of cost variability potentially not clinically warranted was estimated at 33.7%. Given the financial footprint involved, once the findings are extrapolated nationwide, this estimation has far-reaching significance for health funding policy.

## Introduction

### Nature of cost variability

Patient-level cost drivers may be classified as either predictable or non-predictable, based on whether relevant information is available prior to the hospital admission. We propose that predictable cost drivers contain information independent of the care provided in hospital and thus explain warranted variability of patient-level cost of care in hospital. In this scenario, the hospital has no way of easily influencing these factors, with the associated cost variability being ‘pre-determined’ and therefore warranted from the hospital’s perspective. For example, a patient’s prior health status (i.e., co-morbidities), socioeconomic status as well as demographic factors may justifiably lead to varying consumption of health resources while in hospital. As a further example, the widely used DRG (Diagnosis-Related Group) system, which classifies hospital admissions into similar groups, is a variable that links the clinical characteristics of a patient with the expected resource requirements; it can also be viewed as a cost driver of warranted cost variability. Each DRG (e.g., “circulatory disorders without acute myocardial infarction, with invasive cardiac procedure(s), without catastrophic or severe complications”) describes the patient’s condition as well as its severity [1], features that are largely known prior to hospital admission, despite the DRG classification being typically “coded” following completion of the actual episode of care.

Cost drivers that are not known or predictable prior to admission do not relate to the underlying unique patient health predicaments. Such factors may however relate to aspects of patient care provided during hospital admission and may thus contribute to unwarranted (i.e., potentially preventable) variability of cost of care. Large cost variability has been observed between hospitals [2, 3] and it is the unwarranted portion of this variability which may result in no clinical benefit to patients [4]. In fact, large amounts of cost variability with no correlation to the quality of care have been observed previously [5].

The concept of unwarranted nature of hospital cost variability, or equivalently preventability thereof is not yet consistently defined. Useful parallels can be drawn with preventability of patient harm in health care, because harm is known to be strongly correlated with increased cost [6, 7]. Like cost preventability, preventability of patient harm is also inconsistently defined [8]. This suggests a conservative inclination to declaring a particular cost driver as predictable a-priori and thus leading to warranted (or non-preventable) cost variability. Such an approach avoids under-estimating the potential residual unwarranted cost variability, as adhered to in this study.

### Analysis of cost variability

Cost variability in health care—both warranted and not—has been typically modeled with patient-related predictors [9–12] using regression-based risk adjustment methods. There are however two recognised shortcomings of such an approach, which may be why there is no single model yet which is considered as optimal [13, 14]. Firstly, these methods tend to omit some cost drivers that are non-predictable from information available prior to commencement of care. This narrow focus may stem from the imprecise nature of the link between these cost drivers and unwarranted cost variability. Examples of such often ignored cost drivers are: the choice of the admitting and/or discharging unit, the admitting and/or discharging doctor, as well as the timing of the initial clinical encounter as a surrogate for inconsistent (or otherwise) matching of clinical resources to demand, e.g., in after-hours periods. These additional predictors, potentially related to unwarranted cost variability, ought to be included in any model designed to analyse patient-level costs. Secondly, fundamental technical shortcomings of the currently prevailing methods relate to “possible omitted variable biases associated with correlations between unobservable individual specific effects and included covariates” [10]. This is particularly pertinent for patient data which may have many strongly correlated variables that drive costs (e.g., age and co-morbidity).

The first issue is remedied explicitly by the design of this study (see Table 1). A large number of non-predictable cost predictors (not known prior to hospital admission) were included in the model. Clinical judgment was exercised when assigning these variables into either the a-priori predictable or the a-priori non-predictable category, with a general caution to not declare any cost predictor as predictable without sufficient confidence. For example, variables such as day of the week, month, as well as the hour of the day were classified as non-predictable as patients do not plan on presenting at any particular time.

**Table 1:** All potential cost predictors analysed. (See S1 Text).

Potential Cost Predictor Considered	Number of Categories (if applicable)	Predictable (P) or Non-predictable (NP)	Selected as Input for Lasso
age (in years)	N/A	P	Yes
alcohol overuse	2	P	Yes
Charlson Comorbidity Index	11	P	Yes
discharge within last 3 days	2	P	Yes
discharge within last 7 days	2	P	Yes
discharge within last 14 days	2	P	Yes
discharge within last 21 days	2	P	Yes
DRG	617	P	Yes
DVA status	3	P	Yes
IRSAD	N/A	P	Yes
IRSD	N/A	P	Yes
obesity	2	P	Yes
private health insurance status	2	P	Yes
sex	2	P	Yes
smoking	2	P	Yes
admitting medical unit	20	NP	Yes
admitting ward	20	NP	Yes
blood-borne infection(s)	2	NP	No
CHADx Flag	2	NP	No
day of the week	7	NP	Yes
discharging medical unit	25	NP	Yes
discharge ward	15	NP	Yes
doctor at admission	100	NP	Yes
doctor at discharge	100	NP	Yes
fall(s) during admission	2	NP	No
hospital-acquired pressure injury	2	NP	No
hospital length of stay (fractional)	N/A	NP	No
ICU days (discretised)	not set	NP	No
medication errors	2	NP	No
month	12	NP	Yes
time (discetised)	24	NP	Yes
transfer out flag	2	NP	No

While the amount of information is greatly enriched by this inclusion of additional variables, the need to handle correlations between them is potentiated. This is because the possibility of co-linearities increases with the inclusion of additional predictors. This study uses the technique of sparse group *LASSO* (Least Absolute Shrinkage and Selection Operator) to overcome this challenge. A lasso model is one that fits a linear regression via a penalised maximum likelihood [15]. It solves an *l*_1_ optimisation problem to select out unimportant coefficients but to keep the necessary ones in the model. This results in an interpretable model that includes only the pertinent, or principal cost drivers. Moreover, an *l*_1_ method seems appropriate, given its solution exhibits robustness-like properties [16] born out of the fact that it effectively restricts the number of coefficients in the solution, therefore diminishing the effect of outliers. Such resultant relative insensitivity to outliers is beneficial in the case of the skewed hospital cost data [17]. Naturally, there is a balance between including too few and too many predictors. Leaving an insufficient number of predictors in the model may lead to inadequate explanation of the observed cost variability, in turn resulting in a loss of accuracy. On the other hand, too many predictors will also increase error through inclusion of parameters with little additional information. It is therefore convenient that the number of residual predictors left in the lasso model is adjustable and can be set to minimise the resultant error.

The sparse group lasso optimisation is a variation of the more generic lasso technique that accounts for the fact that some variables need to be grouped [18, 19]. This is particularly important when dealing with sparse categorical variables, such as the admitting doctor. The ability to group such variables is important to ensure practical relevance of outcomes, so that each categorical variable can be either discounted or selected as an entire group. The sparse group lasso includes an additional penalty factor in the optimisation in addition to the lasso penalty that encourages such grouping [20, 21]. It is a flexible algorithm that does so with a varying force, as determined by the balance between the two penalty factors (see Eq 1). Grouping more decisively results in selecting out more variables [18] and thus might be expected to decrease the error, but only to a point beyond which the error may increase again due to insufficient number of factors left in the model. Again, the optimal balance is able to be computed based on overall error minimisation considerations.

Regression Penalty in Sparse Group Lasso:

$$\begin{array}{c} \lambda \{(1-\alpha )\sum_{j=1}^{m}\|\beta^{\left(j\right)}{\|}_{2}+\;\alpha \sum_{i=1}^{n}\left|\beta_{i}\right|\} \end{array}$$ (1)

λ  tuning parameter

*α*  grouping parameter

*m*  number of coefficient groups

*n*  total number of coefficients

*β*^(*j*)^  the j-th coefficient group

*β_i_*  the i-th coefficient

### Data pre-processing

Given the large number of data dimensions in our research question, we explored using Principal Component Analysis (PCA) as an initial dimension reducing step. An excessive number of dimensions would make the computing requirements impractical. The use of this technique to pre-process large data is well recognised [22] and it has been specifically used in grouping of binary variables [23]. Hospital-based data is frequently large and can exhibit multi-dimensionality. This leads to high potential error in relation to both the way in which it is measured and recorded. Therefore the PCA is a good choice to pre-process hospital-generated data, as it extracts the most pertinent aspects of the information, leaving out the residual noise. The output of the PCA then represents suitable input into lasso-based analysis (See S2 Text).

The sparse group lasso approach provides values of regression coefficients of the predictors left in the model. Comparison of their absolute values provides estimation of the predictors’ relative importance in terms of explaining patient-level cost variability. In the case of grouped variables, the PCA conveniently converts any group of related variables (e.g., corresponding to each DRG, or each day of the week) into a group with an orthogonal set of elements, i.e., the principal components, which are then analysed by the sparse group lasso method. The orthogonality justifies summation of the absolute values of regression coefficients of all members of each group to accurately estimate the group’s total effect on cost variability via computation of an aggregate coefficient for each group.

## Methods

### Hospital data

Research was undertaken in a public hospital, which is ultimately under the jurisdiction of the Queensland Department (ie Ministry) of Health. The study has been approved by a Human Research and Ethics Committee (HREC), which is the relevant Institutional Review Board. The particular committee is the Royal Brisbane and Womens’ Hospital’s HREC. The Australian approval number is HREC/16/QRBW/61. The Human Research and Ethics Committees in Australia grant approvals in accordance with the Australian National Statement on Ethical Conduct in Human Research (2007), which in turn fulfills the obligations under the Declaration of Helsinki. As the study is only a statistical analysis of large data, with negligible risk of patients being identified, the above-mentioned ethics committee has granted a waiver of patient consent. This waver has been subsequently endorsed and approved by the Queensland Department of Health. The current dates of the ethical approval are from 09 March 2016–09 March 2019.

The data were all hospital admissions to a mid-sized hospital during the 2014/15 financial year. This totaled 53,224 admissions. Some admissions pertain to the same person, with the number of unique patients being 31,449. The hospital serves a geographically contained population and lacks any notable sub-specialisation skews or unusual service gaps which may occur in geographical areas with less defined servicing boundaries (e.g., in a large city with several hospitals in close proximity). This should justify extrapolation and wider applicability of the results.

The potential cost predictors analysed are listed in Table 1. The cost drivers were classified as predictable a-priori and thus representing potentially warranted cost variability, as well as non-predictable, as also depicted in Table 1. This process of classification followed previously used logic [24] and it also incorporated senior medical opinion, in line with the classification principles described above in the Introduction. Predictable drivers were considered as associated with warranted cost variability. Examples of such cost drivers included age and sex. It is specifically worth highlighting that DRG was considered as representing a predictable cost driver a-priori and was classified accordingly. On the other hand, cost drivers such as day of the week or time of the patient’s admission were not reasonably predictable a-priori, and were thus classified as non-predictable, with any related cost variability being potentially unwarranted. We should not expect that a patient admitted on a Wednesday should cost any more or less than a patient with similar characteristics admitted on a Sunday.

### Initial exclusion of factors

**Fig 1:**
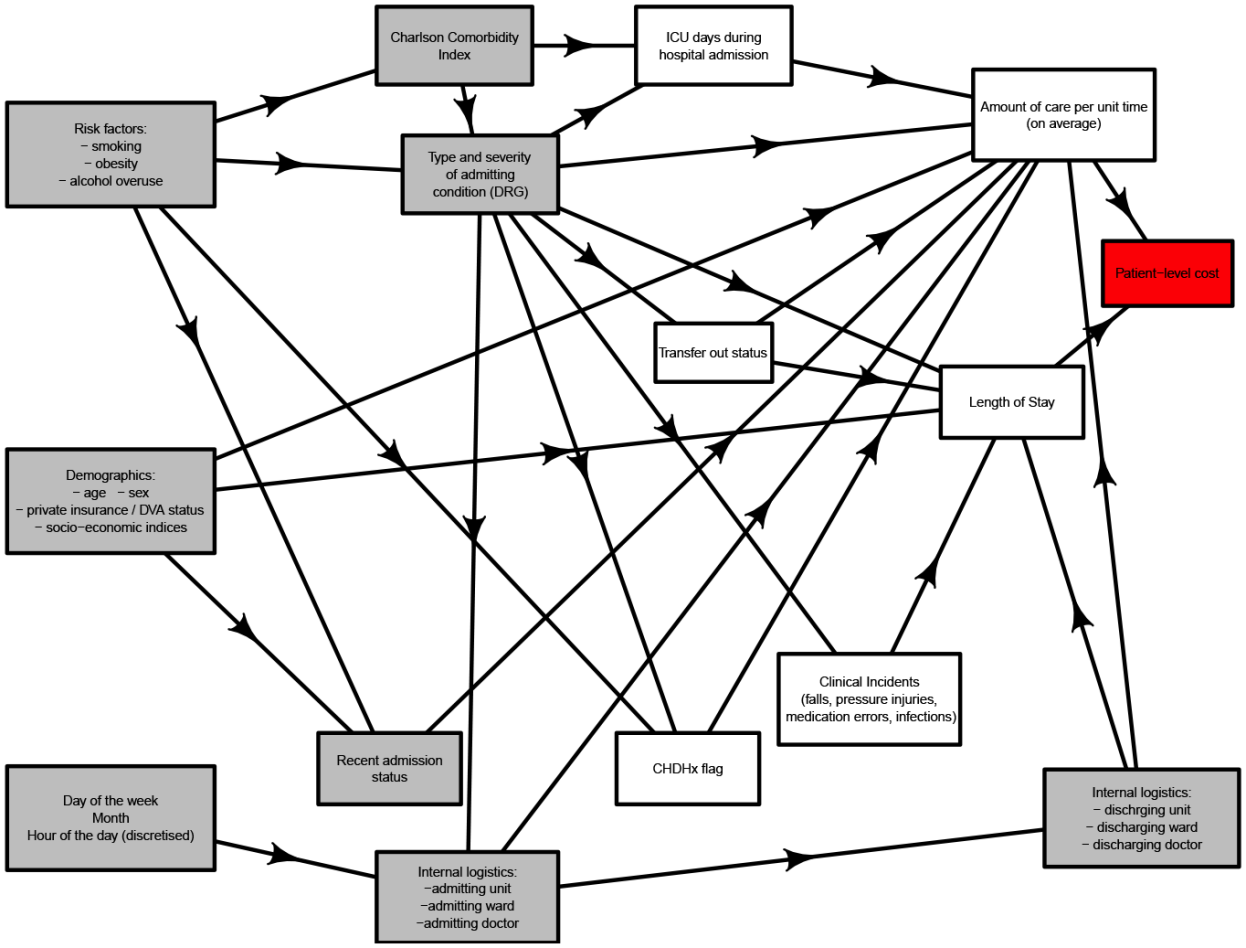
Causal diagram.

Several potential cost drivers available from the hospital information system were then deliberately excluded from further analysis as they were felt to merely represent likely consequences of other variables; see the causal diagram in Fig 1.

The potential driver of days spent in the ICU (Intensive Care Unit) was left out, as it depended strongly on other variables left in the model, in particular the type and severity of the patient condition (already described by DRG), as well clinical decision making (already encapsulated in both the variables of admitting unit and admitting doctor). In fact, the decision to admit to intensive care essentially reflects clinical interpretation of the nature of the patient condition, in the context of limited intensive care resources [25]. The limitation on resources, particularly the immediate availability of intensive care beds is, of course, likely to be more prominent at certain times than at others. While the model specifically includes variables of day of the week, month as well as hour of the day, it is however acknowledged that at least some of this variability is likely random and would not be included therein. Because the very aim at this stage is to identify the proportion of cost variability that is unwarranted and potentially rectifiable, stochastic variability is not modeled in the purposely deterministic model.

There are strong parallels between the decision to transfer a patient to a different facility and to accept a patient to intensive care, as discussed above. Transfer out can occur either because the clinical capacity of the hospital under analysis is thought to be exceeded, because the patient condition necessitates admission under a medical team that is not available locally, or perhaps because the patient condition is considered stable enough for transfer to a lower acuity facility. All these are dependent on clinical decision making within the context of the patient condition. Hence, the potential cost driver of “transfer out” was left out, mirroring exclusion of the variable of intensive care admission.

The flag for selected clinical incidents was left out because clinical incidents were related to other variables left in the model. Clearly, rates of clinical incidents would be necessarily associated with the patient condition (as described by the DRG). For instance, patients with conditions that require multiple medications would be more likely to experience medication-related adverse than those who may take fewer medications. Also, those with medications that are known to decrease blood pressure or cause confusion would be more likely to experience in-hospital falls. Furthermore, medical conditions that limit mobility may render a patient more likely to develop pressure injuries.

There is also likely to be a random component that contributes to when clinical incidents take place. As mentioned above, as the aim at this stage was to identify the proportion of deterministic and thus potentially rectifiable cost variability that is unwarranted, these variables were left out of the model.

Clinical incidents are known to be associated with problems in clinical assessment, communication and decision making, the nature and staff profile of the relevant clinical unit as well as the physical attributes of the surroundings [26]. These are likely to be associated with random events, especially given the emerging theories of accident causation in complex sociotechnical systems [27]. However, any additional deterministic effect would likely be encapsulated by variables of admitting ward, admitting unit, admitting doctor, discharge ward, discharge unit and discharge doctor, which were all left in the model.

Parallels exist between the CHADx flag (which is a flag for hospital-acquired complications) and clinical incidents. Information encapsulated in the CHADx variable is likely to lead to varying DRG assignment and was left out of the model on the basis of the DRG staying in. This is supported by the fact that the CHADx uses routinely abstracted hospital diagnosis and condition-onset information about in-hospital complications [28].

The amount of health care received by patients, when standardised by patient related characteristics is known to be largely driven by clinical decision making [29]. The amount of total clinical care received during an episode of care is however a function of the amount of care provided per unit time, as well as the total duration of the episode of care. Because of inclusion of variables pertaining to doctor as well as the medical unit in the model, both the amount of care per unit time and the length of stay in hospital were therefore excluded from the model. The exclusion of the hospital length of stay (LOS) from the model mirrors other recent work on cost driver analysis in hospitals [24]. Furthermore, the LOS may be also dependent on a wide suite of determinants, some pertaining to patient flow dynamics, rather than to processes related to clinical care.

In summary, the white boxes represent such potential cost drivers, not likely to represent the primary drivers of patient-level costs. This exclusion specifically applied to the hospital length of stay (LOS) and its non-inclusion in the model mirrors other recent work on cost driver analysis in hospitals [24]. The grey boxes represent the variables left in the model, as they were considered to represent, at least in substantial part, primary cost drivers. This preliminary choice of variables does not negate the need to analyse and eliminate correlations between the variables that remain in the model. Accordingly, Table 1 indicates the factors included in the subsequent sparse group lasso analysis, following the causal analysis.

### Subsequent analysis

The CCI (Charlson Comorbidity Index) has been shown to have a predictive association with health care costs [30]. It was obtained from the *Health Roundtable*, an organisation which computes the index for subscribing health providers.

The Index of Relative Socio-economic Advantage and Disadvantage (IRSAD) and the Index of Relative Socio-economic Disadvantage (IRSD) were obtained from the Australian Bureau of Statistics SEIFA 2011 [31] which were added by cross-referencing against the patient’s postal code. Postal codes were missing in just 326 of the 53,224 patients. To prevent loss of other potentially valuable information related to these patients, these were given the average values of both indices, calculated from the remaining data, rather than being excluded.

Other than the postal code data, missing data was negligible. Only one patient had missing data for private insurance (both private insurance as well as DVA or veteran insurance status), smoking, obesity or alcohol flags. All these fields were given modal values, calculated based on all remaining information, in line with the categorical nature of the data.

All categorical variables, except for the CCI, were first represented as sets of binary variables. The relationship of the CCI (ordinal integer values), IRSAD, IRSD and age were first both assumed as linear with respect to the patient hospital costs. These assumptions were felt to be justified in relation to the IRSAD and the IRSD, given the design of both these indices. Appropriateness of these assumptions in relation to the CCI and age was however analysed further with the sparse group lasso re-run, but this time including a squared term for each of these two variables. Computational efficiency necessitated that the re-run was carried out on a random 10% of the data; however it was repeated three times.

PCA was used to pre-process the data and reduce its size without losing vital information. See S3 Text for further description of use of this technique. Output from application of the PCA to categorical groups of factors was used as input into the sparse group lasso. Please see S4 Text on how the optimal lasso parameters of *α* and λ were derived.

Once λ (the lasso penalty) was determined, the corresponding coefficients of the selected variables were used to compare their respective influences on patient-level hospital costs variability. Absolute values of all regression coefficients were first determined, in keeping with the focus being on cost variability rather than the absolute cost values. Absolute values of all coefficients within groups were added together to provide aggregate coefficients for each multi-factor cost driver (e.g., DRG or discharge ward). Each aggregate coefficient is therefore a resultant estimation of the relative contribution of each grouped variable to patient-level cost variability.

Please see the S5 Text for the method used to compare respective effects of categorical and continuous variables. Please see the S6 Text for an explanation of how confidence intervals were constructed. S7 Text describes the computing times associated with the calculations.

### Comparison with simple regression

The results of the sparse group lasso were paralleled by also analysing the data using simple linear regression, for comparison purposes. This used the command *lm*, available in the stats package in *R*. Like sparse group lasso, this analysis was also performed on the data pre-processed by the PCA, to facilitate ease of comparison. Grouping of binary parameter estimates pertaining to the same cost driver and handling of the age, IRSAD, IRSD as well as the CCI coefficients was carried out in a manner identical to the case of the sparse group lasso.

## Results

Table 2 shows the relative significance of the variables included in the model in terms of driving hospital cost variability, by the magnitude of the penalised regression coefficient or the aggregate coefficient (depending on the nature of the cost driver). The values were obtained by first selecting the single optimal λ that minimised the cross-validation error. The optimal λ used in this study was 0.646 (3DP). The predictable cost drivers accounted for 66.3% of the total patient-level cost variability, leaving 33.7% potentially associated with cost drivers not predictable a-priori.

**Table 2:** Results of the sparse group lasso optimisation—Values of penalised regression coefficients.

Cost Predictor Name	Value of the Coefficient or Aggregate Coefficient	95% Confidence Interval	Percentage of Total Variability
age (in years)	59,473	36037–79318	13.44
alcohol overuse	1961	1273–2478	0.44
Charlson Comorbidity Index	6088	4092–7802	1.38
discharge within last 3 days	242	27–422	0.05
discharge within last 7 days	226	37–365	0.05
discharge within last 14 days	324	119–586	0.07
discharge within last 21 days	335	128–573	0.08
DRG	128,148	87,858–175,225	28.96
DVA status	259	128–384	0.06
IRSAD	46,469	26,704–66,915	10.5
IRSD	45,034	27,108–64,053	10.18
obesity	1962	1260–2477	0.44
private health insurance status	577	144–1062	0.13
sex	533	252–810	0.12
smoking	1961	1309–2465	0.44
subtotal			66.34
admitting medical unit	13,792	7806–18951	3.12
admitting ward	19,939	15,498–26,228	4.51
day of the week	0	-	0
discharging medical unit	48,356	39,047–56,229	10.93
discharging ward	66,511	60,783–72,895	15.03
doctor at admission	0	-	0
doctor at discharge	0	-	0
month	0	-	0
time (discretised in whole hours)	313	-484–988	0.07
subtotal			33.66

To illustrate the significance of using the sparse group lasso technique to eliminate the artifacts of co-linearity, Table 3 provides a comparison with results that would be obtained if un-penalised simple linear regression were used instead, as is common practice in hospital cost predictor analysis [24]. We note that the average Variance Inflation Factor related to the coefficients in the linear regression model applied to our data (see Table 3) was 11.4, confirming the existence of significant co-linearity. The variables of alcohol overuse and obesity were found to be exactly co-linear with other variables and thus the corresponding coefficients were not able to be estimated. This is a likely consequence of the fact that both these variables rely on information obtained from codes that contribute to DRG assignment.

**Table 3:** Results of simple linear regression—Values of regression coefficients.

Cost Predictor Name	Value of the Coefficient or Aggregate Coefficient	Percentage of Total Variability
age (in years)	21.4	0.10
alcohol overuse	not estimated	0
Charlson Comorbidity Index	44.9	0.21
discharge within last 3 days	99.2	0.47
discharge within last 7 days	1.1	0.01
discharge within last 14 days	53.7	0.25
discharge within last 21 days	10.0	0.05
DRG	11,518.7	54.18
DVA status	34.0	0.16
IRSAD	43.4	0.20
IRSD	65.6	0.31
obesity	not estimated	0
private health insurance status	21.1	0.10
sex	9.7	0.05
smoking	130.0	0.61
subtotal		56.7
admitting medical unit	994.7	4.68
admitting ward	392.7	1.85
day of the week	31.9	0.15
discharging medical unit	1796.5	8.45
discharging ward	843.0	3.96
doctor at admission	1962.0	9.23
doctor at discharge	2983.0	14.03
month	41.3	0.19
time (discretised in whole hours)	163.3	0.77
subtotal		43.3

*Note*: *the horizontal line between predictors separates the predictable (top) from the not predictable (bottom)*

*Note*: *the horizontal line between predictors separates the predictable (top) from the not predictable (bottom)*

**Fig 2:**
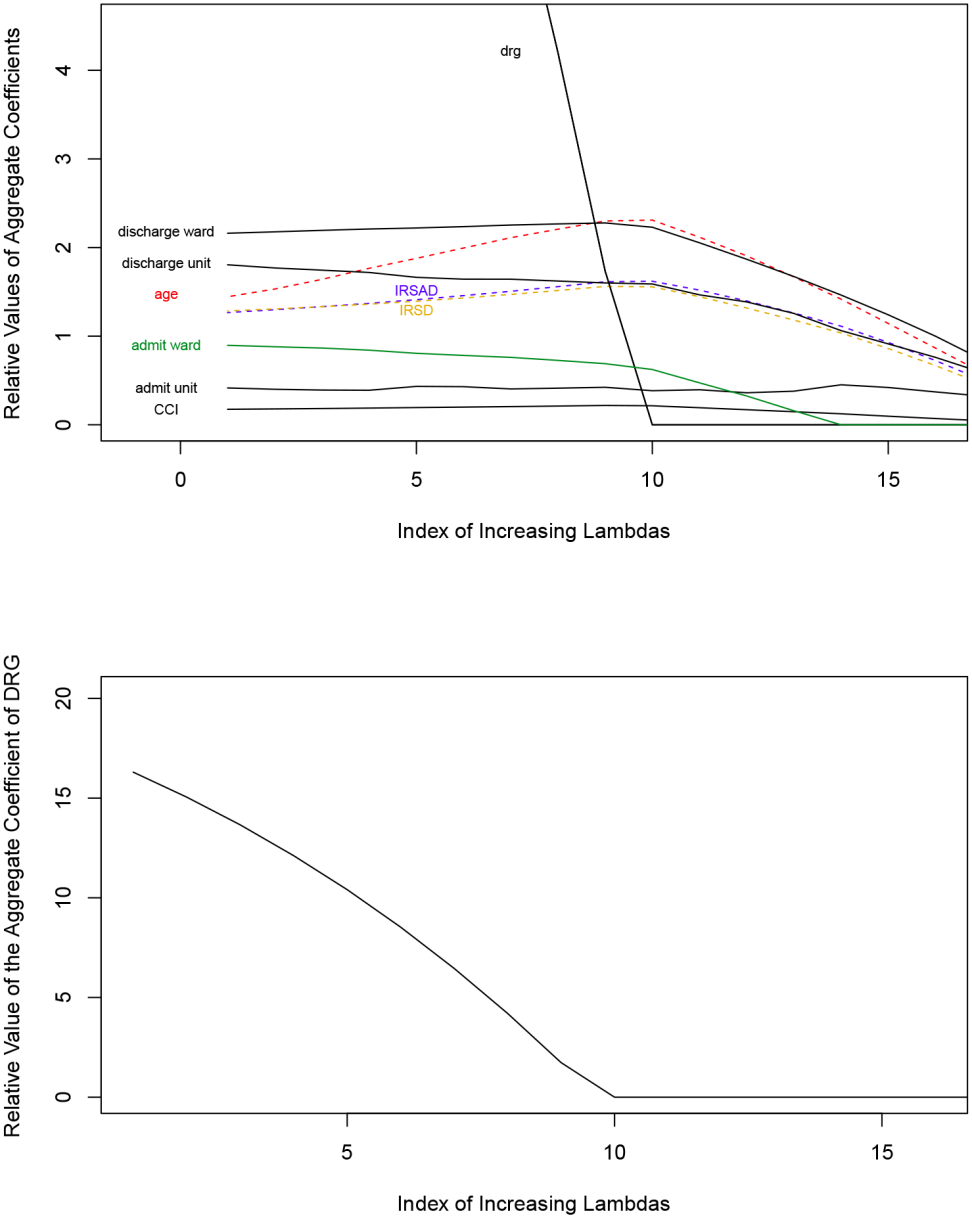
Effect of increasing λ on the magnitude of main coefficients. A: Effect of Varying the Regression Penalty on the Coefficient Size of Major Cost Drivers. B: Effect of Varying the Regression Penalty on the Aggregate Coefficient Size of DRG Alone.

To further illustrate the issue of co-linearity, Fig 2 shows the effect on coefficient values of applying the sparse group lasso to the data, with increasing strength of the sparse group lasso penalty factors (determined by increasing the λ). It is the penalty that effectively converts simple linear regression into penalised regression. The effect of including the penalty (see Eq 1) is sparsity, i.e. ability to select only some groups of variables. The magnitude of the parameter λ determines the strength with which the penalty is applied and therefore the strength of the tendency to eliminate some groups of variables.

**Fig 3:**
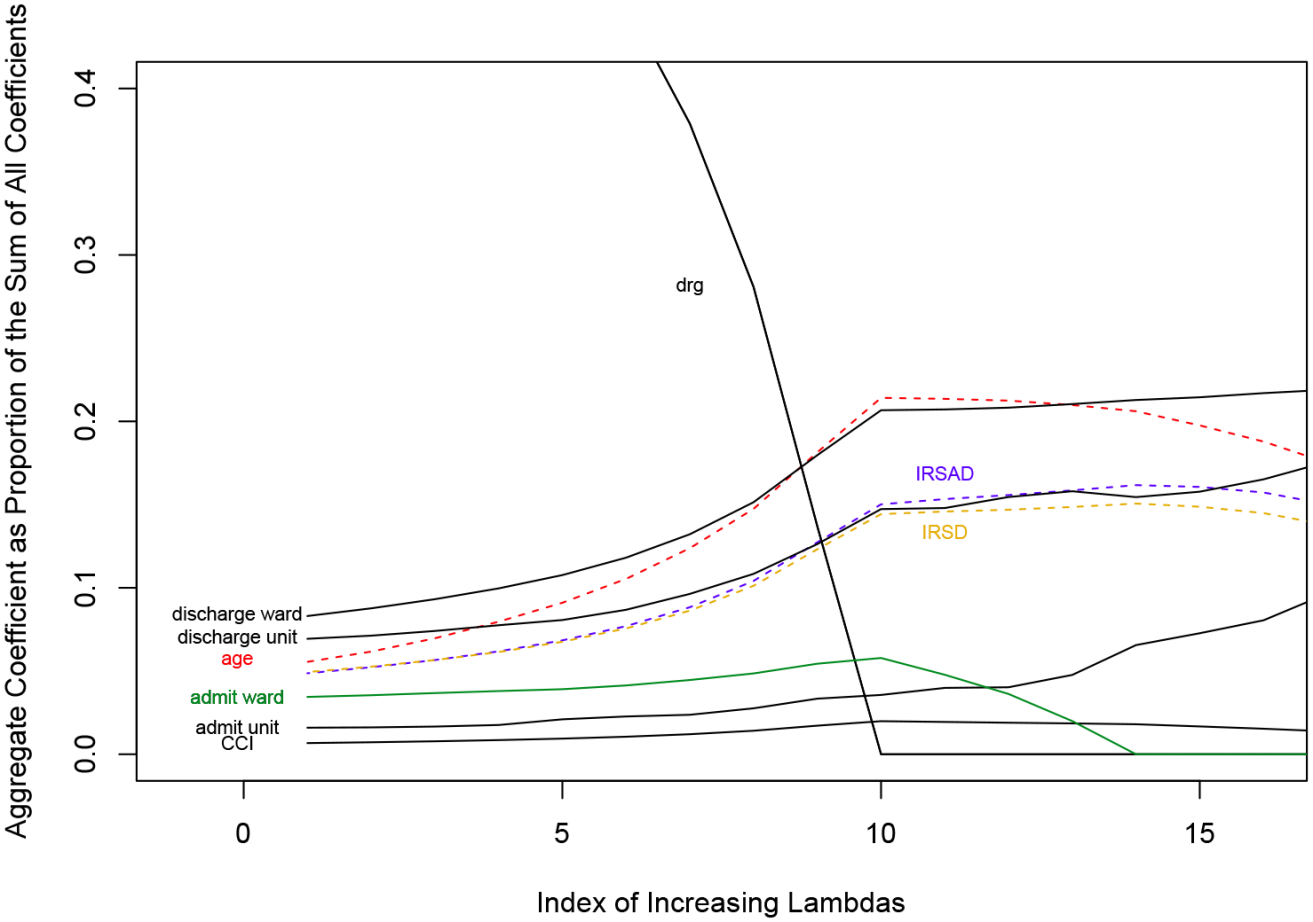
Effect of varying the regression penalty on the relative coefficient size as proportion of all observed variability.

Also, the relative values of the same coefficients or aggregate coefficients as proportion of the overall cost variability are provided in Fig 3, for added clarity. Figs 2 and 3 both serve illustrative purposes and show the coefficients that are numerically most prominent in the proximity of the optimal λ, rather than all the coefficients related to cost predictors. The further to the right, the greater the amount of departure from the simple regression model and the stronger the effect of the sparse group lasso. The proportionally lesser predominance of the DRG as a cost driving factor observed with increasing values of the (co-linearity correcting) λ is the most striking feature in both figures.

The values of the coefficients observed towards the right side of Fig 2A become incongruent with reasonable expectations, as DRG disappears completely as a cost driver. It is reassuring that these observations were found to be associated with increasing cross-validation error. Furthermore, comparing the coefficient values on the left side of Fig 2A (i.e., closest to simple linear regression) and the values in Table 3 (i.e., actual simple linear regression) reveals a necessary steep increase in the values of other variables, predominantly the admitting doctor and the discharging doctor data fields, in the very close proximity to the λ value of 0.

**Fig 4:**
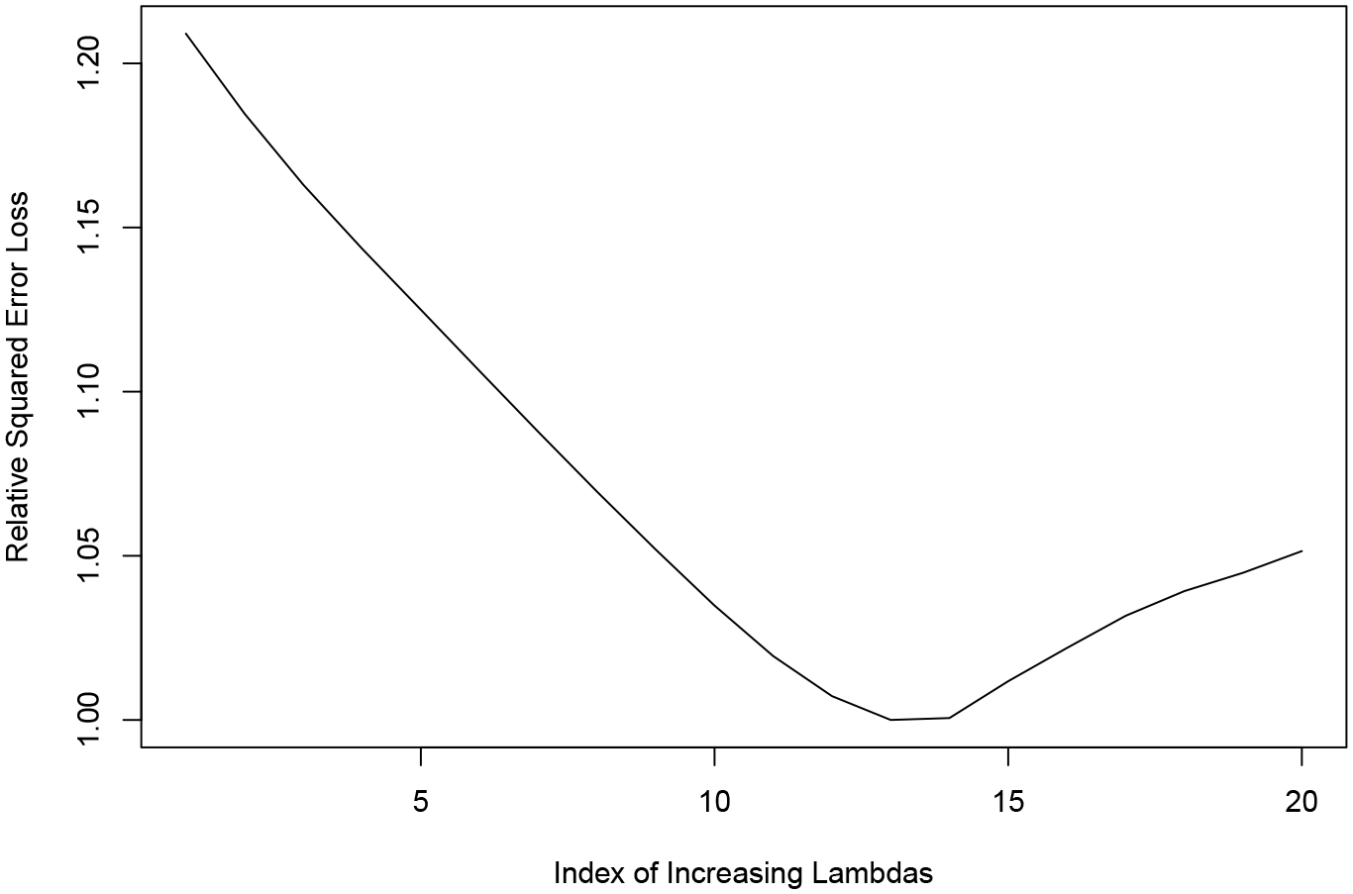
Effect of PCA pre-processing on the cross validation error with varying λ. Pre-processed Data Showing a Clear Minimum.

**Fig 5:**
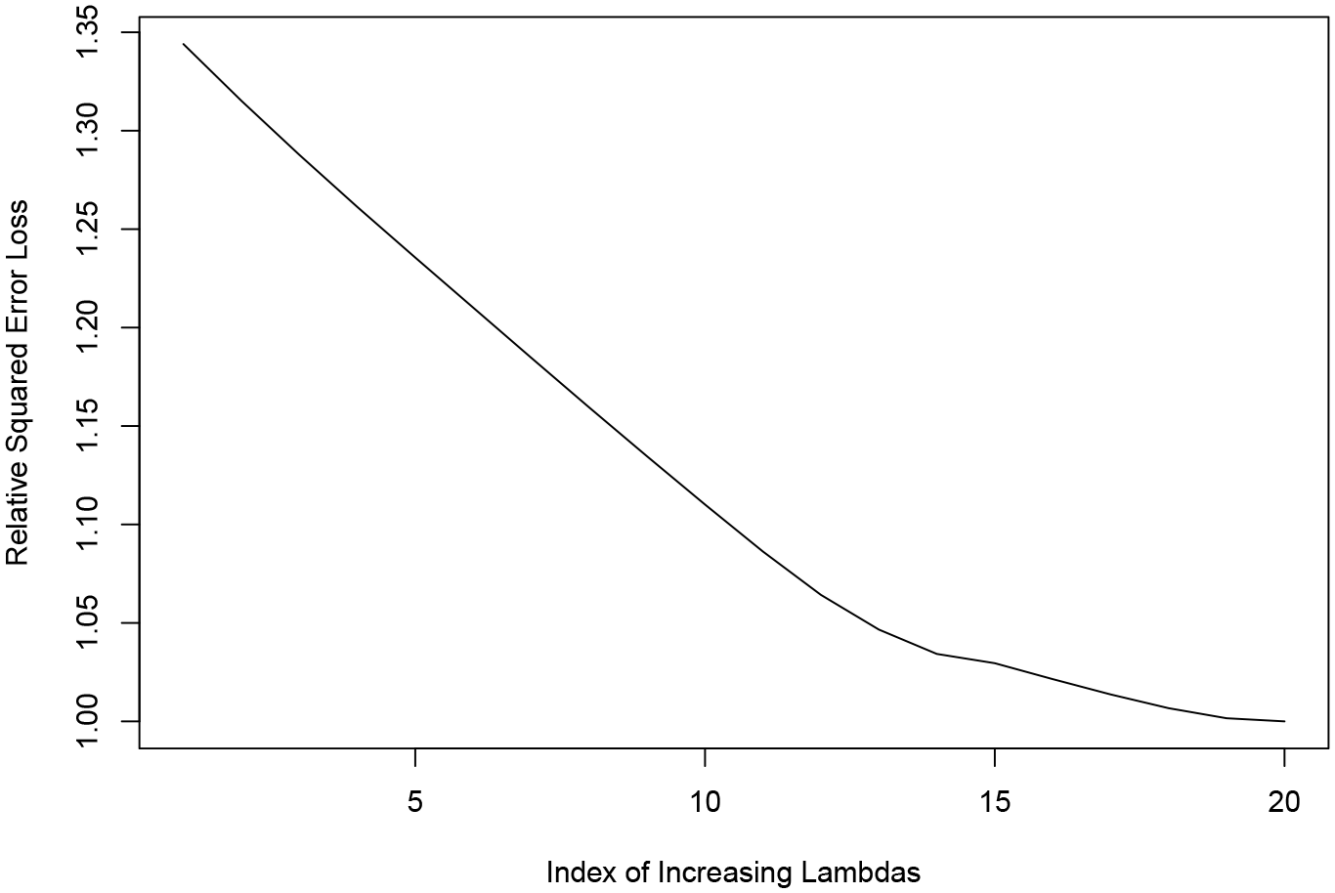
Effect of PCA pre-processing on the cross validation error with varying λ. Data Not Pre-processed and Tending to a Minimum with Maximal Model Sparsity.

The benefit of using Principal Component Analysis to pre-process the data is demonstrated in Fig 4. There is a clear minimum of the cross-validation error observed, coinciding with the optimal λ. This is in sharp contrast to the case without the pre-processing, as depicted in Fig 5, where the residual noise precludes a sensible optimal point with minimal cross-validation error. Both graphs were obtained using the same random 10% sample of the data and both were produced using the same sequence of λ values.

The value of *α* that minimised the cross-validation error was 0.15, i.e., close to maximal grouping. The small value of *α* is consistent with previously published, non-hospital-based research [18], although general caution should be observed in drawing too strong a conclusion here, as consistency across studies may not be automatically expected when the character of the data varies.

We re-ran the sparse lasso, this time relaxing the linearity assumptions with respect to the CCI and the patient age data fields. This tested the suitability or otherwise of relaxing the linearity assumptions of both variables. Adding a squared term to each provides for a wide range of alternative, non-linear relationships with the outcome variable, i.e. patient-level hospital cost. The results demonstrated no significant impact on outcomes for either the CCI or age. Specifically, in each case, the sum of the coefficients of both the linear and square terms were well within the 95% confidence range of the previously obtained coefficients based on linearity assumptions. This indicates that the linearity assumptions were justified.

## Discussion

The study has identified the main drivers of hospital patient-level cost variability. They are: DRG, age, choice of the discharge ward, choice of the discharge medical unit, as well as the two measures of socio-economic status, the IRSAD and the IRSD. The admitting ward, admitting medical unit and the CCI are secondary, albeit still significant cost drivers. Identification of this set of “principal cost variables” is important from a practical perspective as it provides a potential structure for analysis of cost over-runs in a hospital. Any mismatch between budgeted and actual costs could be first broken down by each principal variable and then any problems narrowed down to a particular principal variable could be analysed further, relying on the associations with other non-principal cost variables. This would provide a logical structure for cost analysis which follows the empirically-derived cost architecture. Crucially, such analysis could also be replicated longitudinally, facilitating comparability across time periods and monitoring effects of any cost containment initiatives.

As summarised in Table 2, potentially up to 33.7% of all variability is not determined by patient factors known at the time of the patient’s initial contact with the hospital, and is thus potentially unwarranted. This figure therefore represents potential waste; i.e., it is the upper bound for the waste estimate. However, because of the comprehensive selection of potential cost predictors, there should be good proximity between the upper bound of the unwarranted variability and the actual amount of unwarranted variability. The list of potential cost predictors listed in Table 1 corresponds to previously reported results [24] indicating that socio-economic, demographic and health status variables constituted the main explanatory cost variables, all of which were included here.

The choice of the sparse group *LASSO* model is based on the fact that it is an example of models known to encourage sparsity [32]. Furthermore, the sparse group version of the lasso model is used specifically, based on the belief that although many of the predictors of patient-level cost in hospital are logically grouped, only a few predictors in each group may play a significant role. Sparsity of statistical models is known to help recover the underlying signal in a set of data in exactly such circumstances [33].

The technique of pre-processing large data with the PCA has been demonstrated as a crucial step in the analysis, enabling it to be conducted by extracting pertinent information from the original dataset. This carries wider implications for analysing hospital-generated large data that may be subject to both measurement and input error.

The observed differences between the relative magnitudes of the coefficients / aggregate coefficients in the *LASSO* and linear cases are large. Therefore, the benefit of adequately addressing the inaccuracies stemming from co-linearity of predictors is obvious. In fact, the fundamental difference in results obtained from the co-linearity remedying sparse lasso-based technique as opposed to previously used models that do not adequately address this prominent phenomenon (e.g., simple linear regression) makes it imperative that further research in this field analyses the overlap between potential patient-level cost drivers and uses techniques to remedy the otherwise misleading effects of co-linearity. Our findings may also call into question any previous decisions made on the basis of cost driver identification models. As a minimum, researchers should at least test for the presence of co-linearity using the (intuitive) variance inflation factor (VIF).

Variation between the practice of individual medical practitioners if often proposed as the reason for unwarranted cost variation. It is therefore intriguing that, despite featuring prominently in the simple regression case, consultant (i.e., attending) doctor—both at admission and at discharge—are not significant lasso cost predictors. These variables are excluded readily by the sparse group lasso method, most likely reflecting the importance of a team-based nature of health care delivery, rather than individual practices. One could argue that in complex systems such as hospitals, characterised by high usage of technological aids, it is the sum of interactions between all members of the treating team as well as with non-human agents that is more significant in terms of patient care and thus patient-level cost outcomes than doctor variability. This is reinforced by greater prominence of the variables of discharge ward and discharge medical unit in the lasso results, that perhaps better reflect these complex interactions. Useful parallels may be drawn with the view of health care as a complex socio technical system, such that its overall performance cannot be examined by viewing the performance of its components in isolation. This mirrors the theory of distributed situation awareness which explains ergonomic and quality control outcomes in complex systems characterised by multiple interactions between agents, both human and non-human, in preference to situational awareness of any one agent in isolation [27]. The practical consequence of our findings is that when trying to combat unwarranted variation in clinical practice, and therefore cost, it may be more productive to focus on the functionality of a clinical unit, rather than focusing exclusively on individual practitioners. It may, for example, suggest pursuit of strategies such as inter-disciplinary care protocols or enhancing the micro-culture of a clinical unit in preference to benchmarking individuals.

Use of the lasso method decreases the contribution of DRG as a driver of cost variability to 29.0% from 54.2% in the simple linear regression. The compensatory emergence of discharge ward, discharge medical unit, age, the IRSAD and the IRSD as significant cost drivers following the sparse group lasso analysis (Fig 3) may perhaps appear a surprising result at first, given that the purpose of the DRG system is to identify group of patients with similar costs. However, lack of complete homogeneity of patient costs within a particular DRG [34] is the likely reason for the results obtained under the *LASSO* regularisation. This illustrates the imperfect nature of the DRG as a sole costing tool; and is a likely reflection of the lack of complete homogeneity of all episodes of care classified within a particular DRG. Moreover, this point also illustrates why the focus of analysis in this paper has been on groups of related factors rather than on individual factors: it is important to know how much cost variability is associated with different diagnostic groups (as opposed to—for instance—patient age or day of the week of presentation to hospital) rather than with a particular DRG. DRG assignment is based on the approximately 68,000 ICD 10 codes, as classified by the World Health Organization. It evolves all the time but the contribution of the entire group of DRGs is likely to be more stable over time than that of a particular DRG.

**Fig 6:**
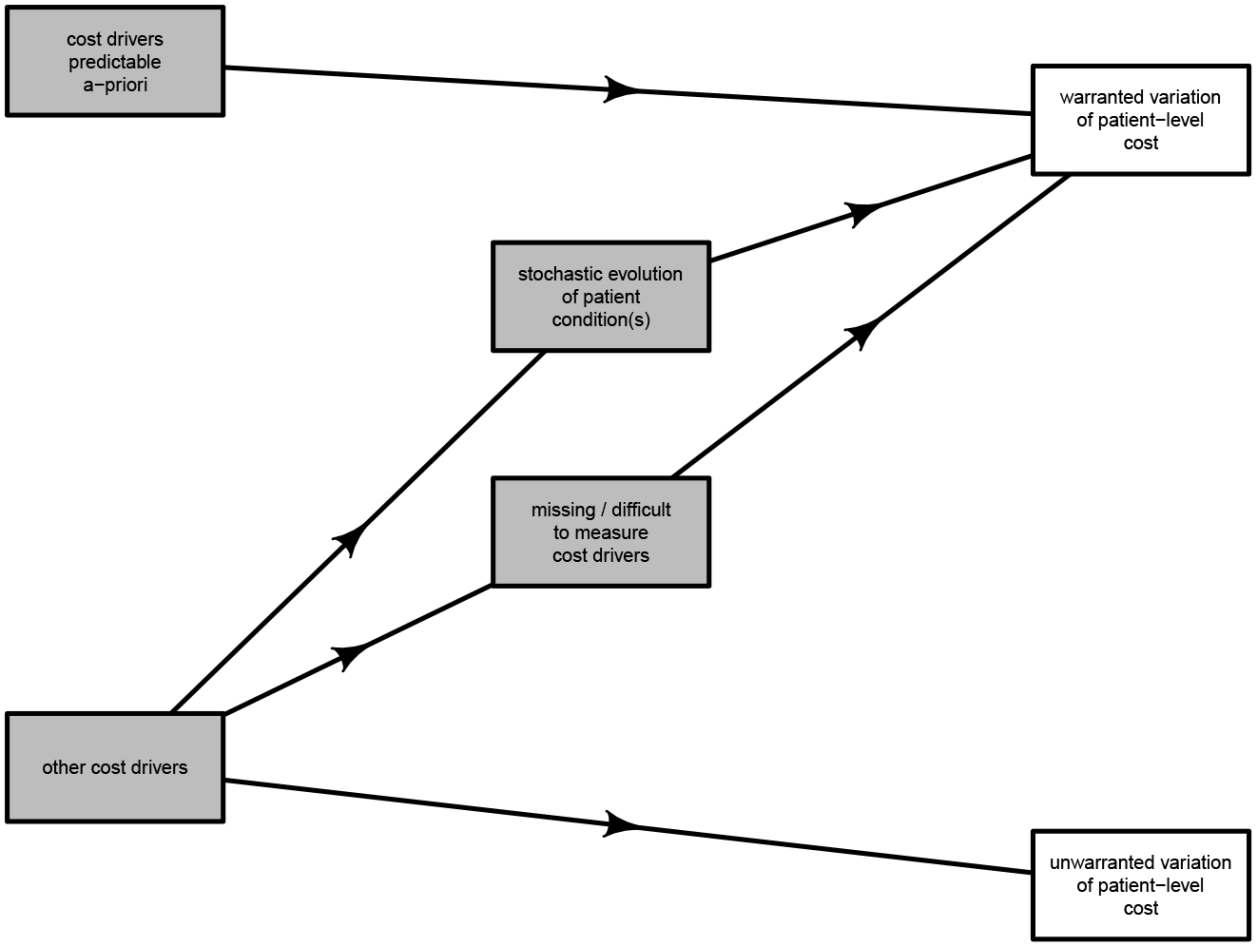
Relationship of warranted and unwarranted cost variation to cost predictors.

From a conceptual viewpoint, the apparent large amount of non-predictable variability of patient-level hospital costs may of course be related to several causes (Fig 6). Firstly, such variability may be a reflection of the fact that some important predictors were still not included. We consider that to be an unlikely explanation here, given the extensive inclusion of potential factors and benchmarking our predictor selection with other work [24]. Secondly, the variability may be due to unwarranted variation in clinical practice and evidence indeed suggests that this is a likely explanation, at least in part [3]. Finally, some of the non-predictable variability may be due to the stochastic nature of the evolution of patients’ conditions, after admission to hospital. Experience from other sectors, such as the financial derivative pricing industry, suggests that inclusion of explicit stochastic elements in predictive models may result in greater accuracy in value estimates [35]. Use of such models that make better allowances for the stochastic, or random behaviour of hospital costs would be in line with the occurrence of unpredictable clinical events [6] as well as the previously reported inability to explain the extent of the observed variation in care [36, 37]. Given the extent to which randomness is encountered in epigenetic variation [38], it is entirely reasonable to extrapolate the concept to human disease and pursue development of an innovative stochastic model that envisages a proportion of patient-level costs being not related to any identifiable cost driver.

The significant presence of non-predictable (and thus potentially unwarranted) sources of cost variability should be of major concern to funders of hospital services. If extrapolated, even if only a proportion of it were actually related to unwarranted variation in clinical practice, the potential saving to the national expenditure would be extraordinarily large. Given these far-reaching conclusions, the analysis would benefit from being replicated at other hospital sites. Furthermore, if confirmed, a model for predicting future health care costs could be developed based on these findings. Such a predictive model, perhaps including allowances for stochastic variability to account for different patients responding differently to treatment, is likely to be more accurate than the currently used methods that tend to rely solely on linear regression techniques. There is therefore room for further research to build on the results obtained herein in estimating the upper bound for waste. It would be highly beneficial to investigate the magnitude of the gap, if any, between the upper bound and the actual amount of waste. This could include the use of simulation (as part of the suite of techniques known as Approximate Bayesian Computation) to reproduce the actual distribution of hospital costs, more accurately estimating the amount of randomness. As natural randomness is not predictable, this would further reduce any estimate of the actual waste. This further work could also better contextualise the practice of setting future efficiency targets based on benchmarks derived from observed costs, as the random nature of the cost distribution may render such targets as unrealistic.

## Conclusion

This article describes a practical method for estimating the drivers of hospital costs and patient-level cost variability. It applies well known and validated statistical techniques of Principal Component Analysis and Sparse Group Lasso to overcome the challenges of multi-dimensionality and co-linearity, respectively. These challenges are commonly encountered with hospital-generated large data and otherwise prevent accurate cost driver estimation. In fact, predicting expenditure in hospitals has long been challenging, with prevalent models producing sub-optimally estimated true costs [39]. While the current findings pertain to a single hospital, they are generalisable due to the ‘standard’ profile of the hospital. The technique itself is also readily adaptable to any other site where accurate cost driver analysis would prove beneficial.

Once estimated, the drivers can be classified as either warranted or unwarranted sources of cost variability. The proportion of variability attributed to unwarranted variability appears rather large. This is postulated as an estimate of the potential waste that could, at least theoretically be minimised. If the findings were further substantiated, the policy implications would be large, perhaps shifting the hospital funding policy focus from matching the cost drivers by appropriate levels of funding to more decisively driving greater care standardisation and thus containing the unwarranted variability. Findings also suggest that clinical care standardisation may be best achieved by focusing on systems of care delivery rather than clinical practice of individuals.

## Supporting information

S1 Text Additional details for Table 1. (PDF)

S2 Text Further details on data pre-processing. (PDF)

S3 Text Further description of principal components analysis. (PDF)

S4 Text Selection of the optimal lasso parameters. (PDF)

S5 Text Method to compare respective effects of categorical and continuous variables. (PDF)

S6 Text Construction of confidence intervals. (PDF)

S7 Text Computing times. (PDF)

S8 Text Documentation for running and installing the software. (DOC)

S9 Text Computer code in *R* to compute the optimal sparse group lasso fit. (R)

S10 Text Computer code in *R* to compute the error-minimising λ. (R)

S1 Fig Test dataset in *Microsoft Excel*. (XLS)

S2 Fig Test dataset in the *txt*. format. (TXT)
