# Supplementary material for: Identification of hospital cost drivers using sparse group lasso
Source: PLoS One. 2018 Oct 10;13(10):e0204300. doi: 10.1371/journal.pone.0204300 (PMC6179217; doi:10.1371/journal.pone.0204300)
Supplement: S1 Text — (PDF) [file pone.0204300.s001.pdf]

Technical Appendix S1

Additional details for Table 1

- Alcohol overuse and obesity flags were both identified based on coding. The factors were noted as contributing to the health condition of the patient.
- Flags for discharge within 3, 7, 14 and 21 days pertain to recent discharge from the same hospital.
- CHADx is the Classification of Hospital Acquired Diagnoses [1].
- Combination of DRG, IRSAD and IRSD is likely to account for rurality and indigenous status.
- Day of the week, month and hour variables all pertain to the time of initial presentation.
- Doctor on admission and doctor on discharge both pertain to the consultant (or attending) medical officer.
- Transfer out flag pertains to the patient being transferred to another acute facility (a hospital).

References

[1] Trentino KM, Swain SG, Burrows SA, Sprivulis PC, Daly FF. Measuring the incidence of hospital-acquired complications and their effect on length of stay using CHADx. Med J Aust. 2013;199(8):543–7.
