# Supplementary material for: Identification of hospital cost drivers using sparse group lasso
Source: PLoS One. 2018 Oct 10;13(10):e0204300. doi: 10.1371/journal.pone.0204300 (PMC6179217; doi:10.1371/journal.pone.0204300)
Supplement: S2 Text — (PDF) [file pone.0204300.s002.pdf]

Technical Appendix S2

Further details on data pre-processing

All the categorical cost factors were grouped together (e.g., DRGs or days of the week) and each such group was conceptually treated as a collection of related factors that together represented a single cost driver. From the practical perspective of a hospital administrator or funder, at this level of analysis one wants to know how much cost variability is, for example, due to DRG generally as a cost driver, as opposed to one individual DRG (out of say 380 different DRGs). The groups of categorical cost factors that only had two categories listed in Table 1 constituted groups with binary structures. The use of Principal Component Analysis was limited to cost factors within each group that did not have a binary structure. Each such group was therefore converted to a lesser number of (non-correlated) factors, decreasing the number of dimensions. This prepared each group for the subsequent lasso analysis. Please refer to the first component of the regression penalty, corresponding to the j-th coefficient group, in Eq 1. Application of this technique ensured that the eventual computing requirements pertaining to the sparse group lasso were significantly decreased.

It ought to be emphasised that no Principal Component Analysis was used to analyse more than one of the categorical factor groups of factors at a time. The potential correlations between the groups of factors were thus not eliminated prior to application of the sparse group lasso.
