# Supplementary material for: Identification of hospital cost drivers using sparse group lasso
Source: PLoS One. 2018 Oct 10;13(10):e0204300. doi: 10.1371/journal.pone.0204300 (PMC6179217; doi:10.1371/journal.pone.0204300)
Supplement: S3 Text — (PDF) [file pone.0204300.s003.pdf]

Technical Appendix S3

Further description of the principal component analysis

Simple scree-plot analysis, which reassuringly tends to over- rather than under-estimate the number of necessary dimensions [1], was carried out with respect to each group of variables. Aggregation of these analyses determined the number of principal components that ensured 60% data variability coverage as the most appropriate use of the technique in this case. This threshold was applied uniformly to all grouped variables. The PCA-processed data was then analysed using the sparse group lasso, implemented in *R* using the package *SGL* [2]. The package includes provisions for the use of k-fold cross validation to select the optimal number of predictors. The analysis used $k = 10$ (the default).

References

[1] Jackson DA. Stopping rules in principal components analysis: a comparison of heuristical and statistical approaches. Ecology. 1993;74(8):2204–2214.

[2] Simon N, Friedman J, Hastie T, Tibshirani R. SGL: Fit a GLM (or Cox model) with a combination of lasso and group lasso regularization; 2013. Available from: `http://CRAN.R-project.org/package=SGL`.
