# Supplementary material for: Identification of hospital cost drivers using sparse group lasso
Source: PLoS One. 2018 Oct 10;13(10):e0204300. doi: 10.1371/journal.pone.0204300 (PMC6179217; doi:10.1371/journal.pone.0204300)
Supplement: S4 Text — (PDF) [file pone.0204300.s004.pdf]

Technical Appendix S4

Selection of the optimal lasso parameters

The optimal choice of α (where $\alpha \in [0,1]$), the parameter that represents the balance between the lasso penalty and the group lasso penalty factors, was chosen by minimising the 10-fold cross-validation error while varying the α from 0.05 (strong grouping) to 0.95 (little grouping). For computational efficiency, necessitated by our large data set, randomly chosen 10% samples of the same available data without replacement were used. The sparse group lasso protocol was then used with the value of α that minimised the cross-validation error to determine the optimal λ; λ being the tuning parameter that determines the strength with which the appropriately balanced combination of the penalty factors is applied. The parameter λ ranges from 0 (no penalty, corresponding to simple linear regression) to a point where all but one variable (or a single group of variables) have been excluded. The final choice of λ was again determined based on cross-validation error minimisation.
