# Supplementary material for: Identification of hospital cost drivers using sparse group lasso
Source: PLoS One. 2018 Oct 10;13(10):e0204300. doi: 10.1371/journal.pone.0204300 (PMC6179217; doi:10.1371/journal.pone.0204300)
Supplement: S5 Text — (PDF) [file pone.0204300.s005.pdf]

Technical Appendix S5

Method to compare respective effects of categorical and continuous variables

The relative apportionment of the observed cost variability to the effects of the individual cost drivers included in the model necessitated a meaningful comparison between the continuous "per-unit" coefficients of age, IRSAD, IRSD and CCI with other coefficients associated with categorical variables. This was achieved by dividing the pre-sorted values of each of the continuous variables into a higher half and a lower half, to mimic a binary variable structure. Then, the difference between the means of the higher and lower halves was calculated. The resultant difference (expressed as number of units) was then multiplied by the corresponding "per-unit" coefficient obtained earlier from the penalised regression analysis.
