# Supplementary material for: Identification of hospital cost drivers using sparse group lasso
Source: PLoS One. 2018 Oct 10;13(10):e0204300. doi: 10.1371/journal.pone.0204300 (PMC6179217; doi:10.1371/journal.pone.0204300)
Supplement: S6 Text — (PDF) [file pone.0204300.s006.pdf]

Technical Appendix S6

Construction of confidence intervals

The *SGL* package for *R* does not provide confidence intervals for the regression coefficient estimates, reflecting the fact that the lasso only provides variables in the maximum likelihood solution. However, these can to be obtained by other methods. In this case, confidence intervals were estimated by first assuming a multinomial model was applicable to the distribution of the absolute values of the coefficients, including the aggregate coefficients. 10% samples (without replacement) were used but this time the analysis provided complete coverage of the entire dataset, with ten non-overlapping samples being used. Therefore, ten estimates for each coefficient were obtained from the sparse group lasso. The multinomial assumption was then used to model the distribution of the coefficients. These were combined by fitting a Bayesian multinomial model in the *WinBUGS* package (version 1.4.3) which estimated the mean and standard deviation of each parameter accounting for the correlation between parameters [1]. This led to *WinBUGS* providing the final estimates of the means and confidence intervals.

References

[1] Lunn DJ, Thomas A, Best N, Spiegelhalter D. WinBUGS &Ndash; A Bayesian modelling framework: concepts, structure, and extensibility. Statistics and Computing. 2000;10(4):325337.
