# Supplementary material for: Identification of hospital cost drivers using sparse group lasso
Source: PLoS One. 2018 Oct 10;13(10):e0204300. doi: 10.1371/journal.pone.0204300 (PMC6179217; doi:10.1371/journal.pone.0204300)
Supplement: S7 Text — (PDF) [file pone.0204300.s007.pdf]

Technical Appendix S7

Computing times

The Queensland University of Technology High Performance Computing (HPC) facility was used to perform the key calculations. Each 10% of data analysed in order to determine the optimal α took approximately 60 hours of computing time, using the cross-validation protocol of the *SGL* package (using the *cvSGL* command). Implementing the sparse group lasso protocol to fit the model took approximately 70 hours of computing time per each 10% data sample. The same command used with the entire data set took considerably longer, at over 330 hours.
