# Supplementary material for: Identification of hospital cost drivers using sparse group lasso
Source: PLoS One. 2018 Oct 10;13(10):e0204300. doi: 10.1371/journal.pone.0204300 (PMC6179217; doi:10.1371/journal.pone.0204300)
Supplement: S8 Text — (DOC) [file pone.0204300.s008.doc]

**Documentation for running and installing the software:**

R can be downloaded – free of charge - from the following website:

<https://www.r-project.org/>

The associated free interface, called R Studio can be downloaded from the following website:

<https://www.rstudio.com/products/rstudio/download/>
